# Reduction of anti-malarial consumption after rapid diagnostic tests implementation in Dar es Salaam: a before-after and cluster randomized controlled study

**DOI:** 10.1186/1475-2875-10-107

**Published:** 2011-04-29

**Authors:** Valérie D'Acremont, Judith Kahama-Maro, Ndeniria Swai, Deo Mtasiwa, Blaise Genton, Christian Lengeler

**Affiliations:** 1 Swiss Tropical and Public Health Institute, Socinstrasse 57, 4002 Basel, Switzerland; 2 University of Basel, Basel, Switzerland; 3 City Medical Office of Health, Dar es Salaam City Council, United Republic of Tanzania; 4 Ministry of Health and Social Welfare, United Republic of Tanzania; 5 Ifakara Health Institute, United Republic of Tanzania

## Abstract

**Background:**

Presumptive treatment of all febrile patients with anti-malarials leads to massive over-treatment. The aim was to assess the effect of implementing malaria rapid diagnostic tests (*m*RDTs) on prescription of anti-malarials in urban Tanzania.

**Methods:**

The design was a prospective collection of routine statistics from ledger books and cross-sectional surveys before and after intervention in randomly selected health facilities (HF) in Dar es Salaam, Tanzania. The participants were all clinicians and their patients in the above health facilities. The intervention consisted of training and introduction of *m*RDTs in all three hospitals and in six HF. Three HF without *m*RDTs were selected as matched controls. The use of routine *m*RDT and treatment upon result was advised for all patients complaining of fever, including children under five years of age. The main outcome measures were: (1) anti-malarial consumption recorded from routine statistics in ledger books of all HF before and after intervention; (2) anti-malarial prescription recorded during observed consultations in cross-sectional surveys conducted in all HF before and 18 months after *m*RDT implementation.

**Results:**

Based on routine statistics, the amount of artemether-lumefantrine blisters used post-intervention was reduced by 68% (95%CI 57-80) in intervention and 32% (9-54) in control HF. For quinine vials, the reduction was 63% (54-72) in intervention and an increase of 2.49 times (1.62-3.35) in control HF. Before-and-after cross-sectional surveys showed a similar decrease from 75% to 20% in the proportion of patients receiving anti-malarial treatment (Risk ratio 0.23, 95%CI 0.20-0.26). The cluster randomized analysis showed a considerable difference of anti-malarial prescription between intervention HF (22%) and control HF (60%) (Risk ratio 0.30, 95%CI 0.14-0.70). Adherence to test result was excellent since only 7% of negative patients received an anti-malarial. However, antibiotic prescription increased from 49% before to 72% after intervention (Risk ratio 1.47, 95%CI 1.37-1.59).

**Conclusions:**

Programmatic implementation of *m*RDTs in a moderately endemic area reduced drastically over-treatment with anti-malarials. Properly trained clinicians with adequate support complied with the recommendation of not treating patients with negative results. Implementation of *m*RDT should be integrated hand-in-hand with training on the management of other causes of fever to prevent irrational use of antibiotics.

## Background

One essential component of the global malaria control strategy is prompt diagnosis and treatment (within 24 hours of onset of illness) with an effective drug [1]. Because of the scarce availability of laboratory facilities and the high mortality of malaria in young children, presumptive treatment in case of fever was seen as the only practical solution to improve child survival. This strategy thus became part of the Integrated Management of Childhood Illness (IMCI) decision chart. The strategy of presumptive treatment was easily and rapidly adopted by health workers to such an extent that it started also to be applied beyond the initial high-risk group: 1) to children older than five years and even adults; 2) in low endemicity areas; and 3) in setting where laboratory diagnosis was actually available [2]. This led to a situation in which the principle of proper diagnosis prior to treatment became an exception rather than the rule. Whatever the medical history (when taken) and irrespective of the clinical examination (if done at all), the same treatment is prescribed: an anti-malarial drug, possibly supplemented by an antipyretic. When the patient returns with persistent fever a second-line anti-malarial drug is given, sometimes intravenously. This may go on until either the spontaneous recovery of the patient from his/her (often viral) illness or up to a deterioration of the patient's condition due to an unrecognized bacterial infection. The strategy of presumptive treatment of all fevers with anti-malarials leads clinicians to believe that all fevers are due to malaria, resulting in a massive over-diagnosis [3,4], and more importantly to ignoring non-malaria causes of fever that have similar, or even higher case fatality rates than malaria [5,6].

The availability of reliable, easy-to-use and affordable malaria rapid diagnostic tests (*m*RDTs) allows now a realistic switch from presumptive treatment to laboratory-confirmed diagnosis and treatment upon result [7]. This is especially important considering the trend of malaria decline in Africa, which leads to a strong reduction in the proportion of fevers due to malaria [8]. There is general agreement that diagnosis should be part of fever case management and WHO has just changed its recommendation from presumptive to laboratory-based diagnosis and treatment upon result [9]. Hence, the discussion is now no more on whether laboratory diagnosis for malaria should be deployed [10], but on how best to implement it. Zambia was the first sub-Saharan country to deploy *m*RDT at the national scale in 2004 followed by Senegal in 2006. Since then, several countries have adopted laboratory-confirmed diagnosis, even in highly endemic areas. However, the implementation of *m*RDT at scale poses many challenges. Rigorous procedures to train and supervise clinicians, to strengthen procurement systems and to ensure quality assurance need to be established. Strong monitoring and evaluation plans need to be put in place. The impact of large-scale implementation of *m*RDT needs to be carefully assessed in different settings and health systems to ensure that it actually reduces over-diagnosis, wastage of anti-malarial drugs and prevents patient suffering.

In order to evaluate the impact of *m*RDT implementation on anti-malarial use and fever case management, a large study was conducted under near-programme conditions in Dar es Salaam, the main urban setting of Tanzania with low to moderate malaria endemicity. The primary objective was to measure the change in overall anti-malarial consumption at different levels of the health system. Secondary objectives were to assess the effect of *m*RDT implementation on the number and type of patients tested and/or treated for malaria, the number of non-malaria laboratory tests performed and the number of antibiotic prescriptions. Health outcomes were not considered since the safety of withholding anti-malarials in febrile children with a negative result for a rapid test was the subject of a rigorous separate assessment published elsewhere [11]. To ensure robustness in the findings, data were collected from two independent sources and effects of *m*RDT implementation evaluated with three different study designs, including a cluster randomized controlled analysis. Introducing *m*RDT represents a new approach to managing patients, and requires thus clinicians' behaviour change. The health facility was therefore used as allocation and randomization unit and not the patient as in previous studies [12-14].

## Methods

### Study setting and population

**Figure 1:**
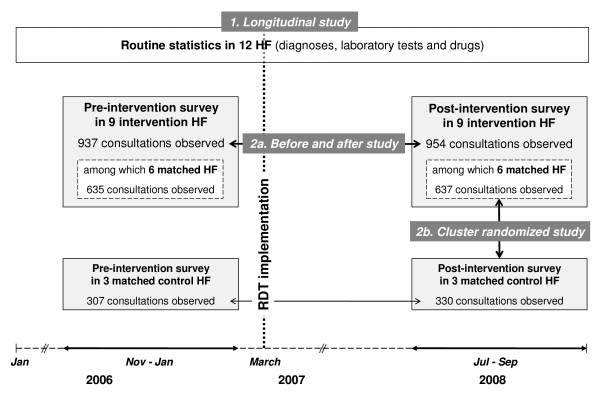
**Study methodology with 3 different evaluation methods: **(1) longitudinal routine statistics collection, (2a) repeated cross-sectional surveys on consultations processes with a before-and-after comparison and (2b) cluster randomized controlled comparison of consultation processes. HF = Health facilities.

The study took place from January 2006 to September 2008 in Dar es Salaam, the economic capital of Tanzania, with an estimated population of over 3,000,000 inhabitants. The level of malaria endemicity has been rather low since at least 2003 [15], with an entomological inoculation rate of 1.3 and a parasite prevalence in the general population of 7% in 2007 [16]. Transmission is perennial with peaks during the main rainy season, which lasts from March to May. Dar es Salaam has three municipalities with a public health system organized in three levels (district hospital, health centres and dispensaries). All three district hospitals (Mwananyamala, Amana and Temeke hospitals) were included. In each municipality a trio of similar HC/D (nine in total) was created on the basis of the following criteria: availability of microscopy, accuracy of the general registers (called MTUHA books) and laboratory registers, socio-economic status of the catchment population, quality of governance of the health facility (HF) and willingness of the staff to participate. One HC/D in each trio was then randomly assigned to be the control HF. There were thus six intervention HF (two HC/D per municipality) and three control HF (one HC/D per municipality); the intervention was also implemented in the three hospitals but for which no control could obviously be found (Figure 1).

### Intervention

In February 2007, after a sensitization meeting with the persons in charge of the nine intervention HF, the City Medical Officer of Health (JKM, who was also one of the investigators) and representatives of the Municipal Medical Offices of Health, five one-day training sessions were organized and attended by a total of 116 clinicians, 31 laboratory technicians, 31 nurses and three pharmacists. The training included one hour on the situation of malaria in Tanzania, half an hour on malaria diagnosis in Africa and in Dar es Salaam, one hour on the clinical use of each type of malaria tests, one hour practical in which participants performed a *m*RDT on each other, and finally two hours of group work on five clinical case studies. The guidelines for the use of *m*RDTs were the following: 1) only patients complaining of fever should be tested; 2) no anti-malarials should be prescribed when the result of the *m*RDT is negative, regardless of the age of the patient; 3) for non-malaria problems, IMCI guidelines should be followed in children under five years.

Investigators then went to each HF to discuss *m*RDT implementation using a standard check-list to be filled in by the focal person for *m*RDT. Between mid- and end of March 2007, the first consignment of *m*RDTs was brought to each HF and a supervision visit conducted 3 days later. Thereafter, supervision took place 1, 2, 5, 10 and 15 months after *m*RDT introduction. Specific problems in 4 HF were addressed by one or two additional on-site meetings. Importantly no incentives were given to any health worker. Control HF were given *m*RDTs after 18 months (November 2008), after a similar training of their clinical staff.

### Project design

In order to come to robust conclusions data were collected with two complementary tools, and outcomes assessed with three different designs (Figure 1).

#### Data collection tool 1: routine statistics from ledger books

A longitudinal study based on the continuous monitoring of routine statistics (MTUHA books) was conducted in intervention and control HF for a period of 15 months before (including one long and one short rainy season) and 18 months after *m*RDT implementation (including two long and one short rainy seasons). All MTUHA books from the years 2006 to 2008 were collected to get monthly information on the following: number of new attendances, number of specific diagnoses, number of laboratory tests, number of malaria tests and their results. The number of anti-malarials and antibiotics consumed per month per health facility were collected from ledger books in the main stores. To get the monthly consumption for each drug, the total number of tablets, vials or blisters (for artemether/lumefantrine - ALu) issued by the main store to the different departments of the HF was counted, excluding the drugs issued to another HF. To get the monthly number of ALu dispensed to patients, the number of patients receiving one of the four types of blisters was counted from the books used at the dispensing windows.

#### Data collection tool 2: cross-sectional surveys of consultation processes

Two cross-sectional surveys were also conducted, one that took place 2-5 months before and the other 15-18 months after *m*RDT implementation in the nine intervention and three control HF (Figure 1). In each HF, 100 consultations were observed at the Outpatient Department by a person (clinical officer or nurse) that was independent from the health facility and from the research team and did not know what the primary outcome of the study was. In order to lessen the influence of season, 50 consecutive consultations were observed in each HF in a first week and an additional 50 consultations were observed 6 weeks later. The targeted sample size for each of the two surveys (before and after) was thus 1,200 consultations. The sample size of 600 patients in the intervention and 300 patients in the control primary care HF was not calculated to detect a significant difference on the primary outcome since the effect of the introduction of mRDT on anti-malarial prescription was expected to be considerable and hence the sample size needed very small. But a small sample size would have precluded assessing with sufficient confidence the effect of mRDT introduction on other outcomes of interest. Hence, the sample size was calculated to allow the detection of a 20% difference in secondary outcomes, i.e. proportion of patients tested and proportion of patients prescribed antibiotics (assumed to be both 50% in the intervention group) with a power of 80% and a significance level of 0.05.

The inclusion criteria for attending patients (of any age) were: 1) first consultation for the present problem; 2) absence of severe illness requiring immediate admission or referral; 3) main complaint not being an injury or trauma. As clinicians in Dar es Salaam (and in most places in Africa) tend to consider the diagnosis of malaria even in the absence of fever [2], fever/history of fever was not used as an inclusion criteria, in order to get the full range of situations in which malaria is diagnosed or treated and thus to compare realistically the results of the cross-sectional surveys with the routine statistics. A standardized questionnaire in Swahili language was used by the observers while following the consultation process.

A first consent to participate was requested from the observed clinician after explaining him/her the aims of the study and the conditions of observation (confidentiality, anonymity and no interference). Informed consent was then requested by the observed clinician from each of his/her patients. The following were observed: complaints mentioned spontaneously by the patient, questions asked by the clinician and the corresponding answers by the patient, signs looked for and laboratory tests ordered by the clinician, tests results, diagnoses, drugs and advice given by the clinician.

This cross-sectional information was analyzed using two designs: (1) comparing anti-malarial prescriptions between pre-intervention and 18 months post-intervention surveys in the nine intervention health facilities (thereafter called before-and-after analysis) and (2) comparing anti-malarial prescriptions between six intervention and their three matched controls contemporaneously, during the post-intervention survey (cluster randomized analysis) (Figure 1).

### Statistical analyses

For the routine statistics data, the unit of analysis was the HF rather than the patient, in order to give the same weight to each HF. Linear models were fitted to the monthly number of anti-malarial or antibiotic doses issued and the number of performed diagnostic tests, and results were expressed as a percentage of the pre-intervention mean. Random effects allowed accounting for differences between health facilities. The results were finally expressed as the ratio of numbers post- over pre-intervention (PP ratio). From this, the percentage reductions could be calculated as (1-PP) * 100. For drug variables, 95% confidence intervals (CI) could not be calculated for the individual HF because of the auto-correlated structure of the data, reflecting month-to-month variations in issuing of drugs. For instance, when large drug volumes were issued from the main store on a certain month, there could be a compensatory reduction the next month. Data were entered in Microsoft Excel 2002 and analysed using the STATA version 10 xtreg command.

For the cross-sectional studies (before-and-after and cluster randomized analyses), the unit of analysis was the patient. All patients observed were included in the study population analysis. Since the number of consultations observed in each facility was almost the same, the weight given to each HF was almost identical and this allowed a direct comparison with the longitudinal study. Comparison of proportions was done by calculating odds ratios using a multilevel mixed-effects logistic regression model to account for clustering. Risk ratios (RR) were calculated from the fitted values for each cell of the 2 by 2 tables. P-values (2-sided) were calculated using Pearson א^2 ^statistics. Data were entered in Epi Info version 3.5.1 and analyzed in STATA version 10.

The level of agreement between results given by the two different sources of data (the routine statistics and the repeated cross-sectional surveys) was measured by the Lin concordance-correlation coefficient [17].

## Results

### Routine statistics from ledger books - longitudinal study

Nearly all required data could be retrieved from the MTUHA books with a few exceptions: among a total of 3,960 monthly data points to be collected (10 variables measured in 12 HF during 33 months), only 36 were missing. For the monthly drug quantification, missing data were replaced by the mean of all data of the corresponding pre- or post-intervention period. Missing monthly numbers of consultations were replaced by the mean of the value from the month before and the month after.

#### Impact of mRDT implementation on anti-malarial consumption

The number of ALu blisters issued by the main store of the 9 intervention HF decreased from a total of 20'660 per month to 7'933 per month after *m*RDT implementation. It decreased in each of the 9 HF with PP ratios ranging from 0.04 to 0.63 (Table 1). When using HF as a unit, the overall PP ratio was 0.32 (95% CI 0.20-0.43) corresponding to an overall decrease of 68%. The impact of *m*RDT was stronger in dispensaries than in health centres or hospitals (PP ratio 0.26 versus 0.35 and 0.34). There was also a clear trend when analysing the data by municipality (PP ratios 0.32, 0.22 and 0.41 for Municipality 1, 2 and 3 respectively). When only looking at the last six months of the study to assess the sustainability of *m*RDT implementation the results were even better (PP ratio 0.25, 95% CI 0.13-0.37). In the three control HF, the overall PP ratio using the whole period was 0.68 (95% CI 0.46-0.91) (Table 1).

**Table 1:** Routine statistics of ledger books: average monthly number of patients positive by *m*RDT, and ALu blisters & quinine vials issued by the main store, before and after *m*RDT implementation, in intervention and control health facilities.

Health facility	**Patients positive by *****m*****RDT** Number per monthn	ALu blisters ^£^			Quinine vials ^&^		
		
		Before *m*RDT initiation* Blisters per month n	After *m*RDT initiation^#^ Blistersper month n	Post-intervention blisters as a proportion of pre-intervention PP (95% CI)	Before *m*RDT initiation^$^ Vialsper month n	After *m*RDT initiation^#^ Vialsper month n	Post-intervention vials as a proportion of pre-intervention PP (95% CI)
**Intervention health facilities**							
Hospital 1	495	4560	1326	0.29	3205	1503	0.47
Hospital 2	323	1500	307	0.20	5049	549	0.11
Hospital 3	335	3100	1608	0.52	1747	1048	0.60
Health centre 1	329	3000	1890	0.63	830	272	0.33
Health centre 2	209	1430	268	0.19	553	86	0.16
Health centre 3	93	1540	360	0.23	177	92	0.52
Dispensary 1	43	650	25	0.04	59	26	0.44
Dispensary 2	101	770	202	0.26	245	85	0.35
Dispensary 3	210	4110	1947	0.47	303	111	0.37
Total of 9 HF^Ŧ^				0.32 (0.20 - 0.43)			0.37 (0.28 - 0.46)
Total of 6 matched intervention HF^Ŧ^				0.30 (0.15 - 0.46)			0.36 (0.24 - 0.48)
**Control health facilities**							
Control 1	N.A	1900	1952	1.03	280	766	2.73
Control 2	N.A	3410	1353	0.40	209	151	0.72
Control 3	N.A	4180	2617	0.63	217	871	4.01
Total of 3 matched control HF^Ŧ^				0.68 (0.46 - 0.91)			2.49 (1.62 - 3.35)

^£ ^One blister of ALU is needed for one anti-malarial course, whatever the age or weight of the patient, **^&^**Between 2 and 6 vials are used per anti-malarial course, * observation period of only 3 months because ALu only introduced in Tanzania in January 2007, ^# ^observation period of 18 months, ^$ ^observation period of 15 months, ^Ŧ ^allowing for random-effect.

**Figure 2:**
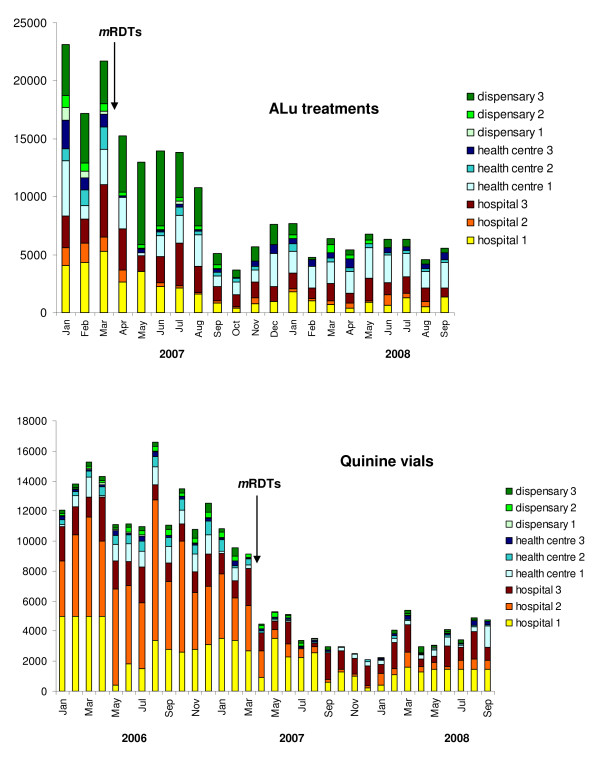
**Number of artemether-lumefantrine (ALu) treatments and quinine vials issued monthly in each of the 9 intervention health facilities**. Pre-intervention follow up times vary because ALu was only introduced in January 2007.

Figure 2 shows the monthly consumption of ALu over time, with the contribution of each HF. The four weight categories of ALu blisters were included, with one blister counting as one anti-malarial treatment course. There was a marked decrease in ALu consumption just after *m*RDT initiation and then a further decrease four months later. The initial four months of the intervention period was used to identify and rectify operational problems (use of microscopy instead of *m*RDT, lack of trust in laboratory technicians, conflict of interest with private laboratories, and reshuffle of staff) in the four HF where little impact was observed during the first four months.

A similar reduction of injectable quinine consumption was found in each of the nine intervention HF (average PP ratio 0.37, 95% CI 0.28-0.46) (Table 1). For quinine there was also a longer period of observation before *m*RDT initiation (15 months) compared to ALu which was only introduced in the country in January 2007. In the three control HF, there was a marked increase in quinine use over the same period (PP ratio 2.49, 95% CI 1.62-3.35).

Table 1 shows that in some HF, the number of issued ALu blisters was much higher than the actual number of positive patients (median excess: 168%). These amounts represent the quantities 'consumed' by the HF and not necessarily the numbers of blisters received by patients. When looking at data from dispensing books after *m*RDT implementation, this excess of quantities consumed over quantities dispensed was confirmed (median excess: 145%). "Mishandling" of drug stocks is likely to be the main reason for the over-consumption of anti-malarials after *m*RDT implementation (although at a much lower level than before).

#### Impact of mRDT implementation on malaria testing

**Figure 3:**
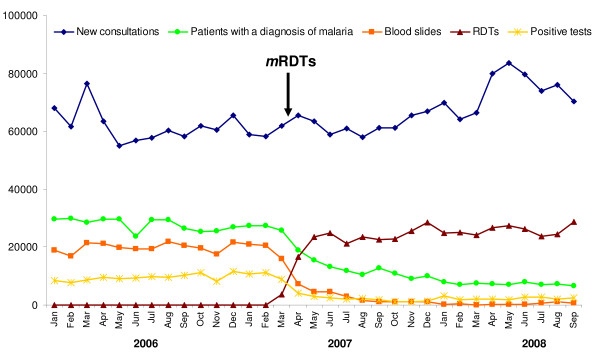
**Number of new consultations, blood slides and *m*RDTs performed, patients with a diagnosis of malaria and positive malaria tests, over time, in the 9 intervention health facilities**.

In the nine intervention HF, from January 2006 to March 2007 (before *m*RDT initiation) a total of 20,143 blood slides were performed on average per month. After *m*RDT initiation, 27,398 *m*RDTs and 768 blood slides were performed on average per month (Figure 3). Although this was not formally recommended during training, microscopy was almost entirely replaced by *m*RDT as first-line malaria test, except for a few special cases (admitted patients, persisting fever in outpatients with malaria, during short periods of *m*RDT stock out). The number of patients attending the nine intervention HF for a new consultation increased slightly over time (61'642 before and 68'065 after *m*RDT implementation on average per month). The proportion of patients tested for malaria increased in all health facilities except 2, from a median of 30% to 42% (PP ratio 1.21) when *m*RDTs were introduced, and thereafter the proportion of patients tested was stable up to the end of the project (Figure 3).

#### Impact of mRDT implementation on diagnoses

The total number of patients with a diagnosis of "malaria" given by the clinicians was 27,693 per month before and 9,920 after *m*RDT initiation. This represents a three-fold decrease (PP ratio 0.33, 95% CI 0.28-0.38) (Figure 3). By contrast, for the diagnoses of acute respiratory infection, pneumonia, diarrhoeal diseases and urinary tract infections there was no change or even an increase after *m*RDT implementation (PP ratio 1.02, 1.29, 1.15, 1.46, respectively). The number of "ill defined syndrome" as well as of "other diagnoses" increased much more (PP ratios 2.14 and 2.36, respectively). In the three control HF, there was no change in the number of patients with a diagnosis of malaria (PP ratio 1.03, 95% CI 0.82-1.24), while acute respiratory infections, pneumonia, diarrhoeal diseases and urinary tract infections increased after *m*RDT implementation (PP ratio 1.59, 1.30, 1.55 and 2.12, respectively).

#### Impact of mRDT implementation on malaria positivity rate

At the time of microscopy, the positivity rate of the routine malaria tests in the nine HF was very high: 49% (range 13 - 88%) and it was similar in the three types of HF: 43% in hospitals, 60% in health centres and 57% in dispensaries. After intervention, the positivity rate of routine *m*RDTs was only 8% (range 6 - 12%)(Figure 3), which is in line with what had been shown in previous studies using expert microscopy in Dar es Salaam [15]. The performance of routine *m*RDTs evaluated in one health centre was excellent (97% sensitivity, 97% specificity) [18].

#### Impact of mRDT implementation on antibiotics consumption

The total consumption of oral antibiotics did not change after *m*RDT implementation (PP ratio 1.02, 95% CI 0.92-1.13). In control HF, the total consumption of all oral antibiotics increased a bit more (PP ratio 1.26, 95%CI 1.00-1.52) than in the intervention HF (PP ratio 1.14, 95% CI 1.00-1.28). In general, the amounts of antibiotics consumed by HF were very high: about 38% of newly attending patients received an oral antibiotic after *m*RDT implementation versus 40% before.

#### Impact of mRDT implementation on laboratory tests other than malaria

The number of urine analysis and direct stool examination increased slightly after *m*RDT implementation (PP ratios 1.18 and 1.23 respectively) in intervention HF. In control HF, both type of investigations increased more (PP ratios 1.66 and 1.74 respectively).

### Cross-sectional surveys: before-and-after analysis

The before-and-after analysis was based on the repeated cross-sectional observation of consultations before and after intervention in nine intervention HF (Table 2). No patient refused to be observed. The proportions of children less than five years, children 5 to 15 years and adults were similar in the pre- and the post-intervention surveys (52%, 14% and 34% versus 53%, 16% and 32%, p = 0.3), while slightly more female patients were included in the pre-intervention survey (60% versus 54%, p = 0.02).

**Table 2:** Before-and-after analysis based on repeated cross-sectional surveys investigating the consultation process: effect of *m*RDT implementation on the main outcomes.

	Before *m*RDT implementation ***Total patients = 937***		After *m*RDT implementation *Total patients = 954*		Risk ratio(accounting for clustering)	
	
	n*	% (95% CI)	n*	% (95% CI)	Risk ratio (95% CI)	*p-value*
*Effect of mRDT implementation on **anti-malarial treatment***						
Patients treated with anti-malarials						
All patients	894	75% (72-78)	912	20% (17-22)	0.23 (0.20 - 0.26)	< 0.001
Patients complaining of fever	755	81% (79-84)	682	24% (20-27)	0.25 (0.22 - 0.29)	< 0.001
Patients not complaining of fever	139	42% (33-50)	230	7% (4-11)	0.16 (0.10 - 0.27)	< 0.001

*Effect of mRDT implementation on **adherence to malaria test result***						
Patients treated with anti-malarials						
Patients with a positive malaria test	370	99% (99-100)	126	99% (98-100)	1.00 (0.98 - 1.01)	0.8
Patients with a negative malaria test	215	53% (47-60)	628	7% (5-9)	0.09 (0.06 - 0.13)	<0.001

*Effect of mRDT implementation on **selection for malaria testing***						
Patients tested for malaria						
All patients	937	68% (65-71)	954	83% (81-85)	1.26 (1.19 - 1.33)	<0.001
Patients complaining of fever	782	71% (68-74)	717	91% (89-93)	1.31 (1.25 - 1.36)	<0.001
Patients not complaining of fever	155	49% (41-57)	237	58% (52-65)	1.21 (0.99 - 1.48)	0.06

*Effect of mRDT implementation on **antibiotic treatment***						
Patients treated with antibiotics						
All patients	894	49% (46-53)	912	72% (69-75)	1.47 (1.37 - 1.59)	<0.001
Patients complaining of fever	755	49% (45-52)	682	73% (69-76)	1.50 (1.38 - 1.63)	<0.001
Patients not complaining of fever	139	52% (43-60)	230	71% (65-77)	1.38 (1.16 - 1.65)	<0.001
Patients with a positive malaria test	370	37% (32-42)	126	35% (26-43)	0.91 (0.68 - 1.21)	0.5
Patients with a negative malaria test	215	54% (47-61)	628	78% (75-81)	1.45 (1.28 - 1.65)	<0.001

*Effect of mRDT implementation on **other laboratory tests than malaria***						
Patient tested for urine infection	937	7% (6-9)	954	13% (11-15)	1.74 (1.31 - 2.31)	<0.001
Patient tested for typhoid (Widal)	937	2% (1-2)	954	1% (1-2)	0.76 (0.36 - 1.63)	0.5
Patient tested for stool parasites	937	6% (5-8)	954	7% (6-9)	1.13 (0.81 - 1.59)	0.5

* numbers differ from total sample size because of the variables applying to different subpopulations of patients (and for drugs because a few patients who did not come back from laboratory to get treatment).

#### Impact of mRDT implementation on anti-malarials prescription

Consultation observations performed before and after *m*RDT implementation revealed a decrease of 77% (from 75% to 20%) in the total number of patients receiving an anti-malarial treatment (Table 2). This decrease (RR 0.23, 95% CI 0.20-0.26) was slightly more pronounced in the subgroup of patients not complaining of fever (RR 0.16) than in the group complaining of fever (RR 0.25). This reduction was mainly due to a drastic change in the adherence of clinicians to test results. At the time of microscopy, 53% (95% CI 47-60) of negative patients were treated with anti-malarials while this proportion was only 7% (95% CI 4-11) after the introduction of *m*RDTs.

#### Impact of mRDT implementation on malaria testing

The overall proportion of patients tested for malaria increased by 26%, from 68% to 83%, RR 1.26 (95% CI 1.19-1.33) after *m*RDT initiation. This increase was mainly seen in patients complaining of fever (from 71% to 91%). In patients without fever, a high proportion was tested after *m*RDT implementation (49% before versus 58% after, p = 0.06) despite training instructions to test only patients with fever.

#### Impact of mRDT implementation on antibiotics prescription

The overall prescription of antibiotics increased after *m*RDT initiation by 47%, from 49% to 72%, RR 1.47 (95% CI 1.37-1.59). This increase was slightly more important in patients complaining of fever (RR 1.50) than in those not complaining of fever (RR 1.38) and was seen in malaria negative patients but not in positive ones. As a result, the vast majority [78% (95% CI 75-81)] of negative patients were treated with an antibiotic after the introduction of *m*RDTs.

#### Impact of mRDT implementation on laboratory tests other than malaria

*m*RDT implementation did not dramatically increase the request for alternative laboratory tests by clinicians, which remained generally low.

#### Convergence of results between the longitudinal and the before-and-after evaluations

**Figure 4:**
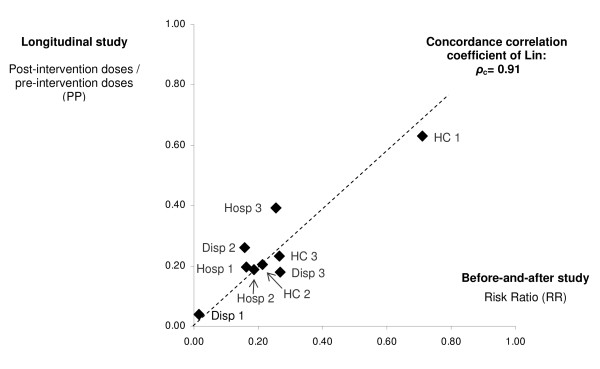
**Association between the measures for first-line anti-malarials consumption reduction by two independent assessments:** (1) health facility routine statistics based on ledger books, and (2) cross-sectional surveys using a before-and-after analysis.

Although the variability between HF was high, there was a strong intra-health facility convergence of the main outcome result (reduction of anti-malarial use) between the longitudinal routine data assessment and the repeated cross-sectional before-and-after assessment (Lin concordance-correlation coefficient: *ρ*_c _= 0.91) (Figure 4). This is a strong confirmation of the robustness of the data.

### Cross-sectional surveys: cluster randomized controlled analysis

The contemporaneous, post-intervention cluster randomized comparison of patient consultations was carried out in six intervention versus three control HF (Table 3). The proportions of children less than five years, children 5 to 15 years and adults were close in the intervention and control groups (39%, 17% and 45% versus 47%, 18% and 35%, p = 0.02), and so was the number of female patients (46% versus 40%, p = 0.1). Outcome results at baseline were similar in intervention and control HF (p > 0.05) (results not shown). The analysis presented below includes mainly data from the post-intervention survey.

**Table 3:** Cluster randomized controlled analysis based on the post-intervention cross-sectional survey investigating the consultation process: comparison between 6 intervention and 3 control health facilities.

		Interventionhealth facilities N = 637	Controlhealth facilities N = 330		Risk ratio(accounting for clustering)	
	
	n*	% (95% CI)	n*	% (95% CI)	Risk ratio (95% CI)	***p-value***
*Effect of mRDT implementation on **anti-malarial treatment***						
Patients treated with anti-malarials:						
All patients	618	22% (19-25)	318	60% (54-65)	0.30 (0.14 - 0.70)	0.007
Patients complaining of fever	473	26% (22-30)	253	65% (59-71)	0.31 (0.16 - 0.67)	0.004
Patients not complaining of fever	145	9% (4-14)	65	38% (26-51)	0.23 (0.10 - 0.55)	0.001

*Effect of mRDT implementation on **adherence to malaria test result***						
Patients treated with anti-malarials:						
Patients with a positive malaria test	96	100% (100-100)	155	100% (100-100)	1	N.A
Patients with a negative malaria test	412	7% (5-10)	128	25% (17-33)	0.09 (0.01 - 0.79)	0.03

*Effect of mRDT implementation on **selection for malaria testing***						
Patients tested for malaria:						
All patients	637	82% (79-85)	330	89% (86-92)	0.93 (0.86 - 1.04)	0.2
Patients complaining of fever	487	90% (88-93)	263	95% (93-98)	0.95 (0.91 - 0.99)	0.03
Patients not complaining of fever	150	57% (49-65)	67	64% (52-76)	0.91 (0.69 - 1.26)	0.5

*Effect of mRDT implementation on **antibiotic treatment***						
Patients treated with antibiotics:						
All patients	618	71% (67-74)	318	53% (48-59)	1.34 (1.08 - 1.70)	0.006
Patients complaining of fever	473	71% (67-76)	253	52% (46-58)	1.44 (1.09 - 1.94)	0.008
Patients not complaining of fever	145	68% (61-76)	65	58% (46-71)	1.17 (0.94 - 1.49)	0.2
Patients with a positive malaria test	96	32% (23-42)	155	28% (21-36)	1.14 (0.77 - 1.66)	0.5
Patients with a negative malaria test	412	77% (73-81)	128	74% (67-82)	1.05 (0.91 - 1.26)	0.5

*Effect of mRDT implementation on **other laboratory tests than malaria***						
Patient tested for urine infection	637	9% (7-11)	330	11% (8-15)	0.73 (0.34 - 1.58)	0.4
Patient tested for typhoid (Widal)	637	2% (1-3)	330	8% (5-11)	0.29 (0.04 - 1.97)	0.2
Patient tested for stool parasites	637	7% (5-9)	330	5% (3-8)	1.27 (0.50 - 3.29)	0.6

* numbers differ from total sample size because of the variables applying to different subpopulations of patients (and for drugs because a few patients who did not come back from laboratory to get treatment).

#### Impact of mRDT implementation on anti-malarials prescription

There was a considerable difference between the two groups in the proportion of patients that were prescribed anti-malarials: 22% in intervention versus 60% in control HF (from a baseline in both of 79%) (RR 0.30, 0.14-0.70) (Table 3). The difference was more pronounced in non-febrile than in febrile patients since only 9% of non-febrile patients received an anti-malarial in intervention HF compared to 38% in control HF (RR 0.23, 95% CI 0.10-0.55). The reasons for the low anti-malarial prescription in intervention HF were multiple: better selection of patients for malaria testing, better specificity of *m*RDT compared to microscopy and better adherence to *m*RDT result. On the other hand, in control HF the lower anti-malarial prescriptions in the post-versus pre-intervention survey was mainly due to a better trust in malaria test results (negative patients treated with anti-malarials decreased from 43% to 25%), even if it was still based on microscopy.

#### Differences between intervention and control HF regarding antibiotic prescriptions

The proportion of patients that were prescribed antibiotics was higher in intervention HF than in controls: 71% versus 53% respectively (RR = 1.34, 95% CI 1.08-1.70) - from a baseline of 50 and 51%. There was, however, no significant difference by category of patients (with/without fever; with positive/negative result), which confirmed that the overall difference in antibiotic prescription was almost only due to the higher number of negative patients and not to a behavioural change of clinicians.

#### Impact of mRDT implementation on laboratory tests other than malaria

The proportions of patients tested for urine, stool or the Widal test did not increase after *m*RDT implementation in both the intervention and control HF (p > 0.05).

## Discussion

**Figure 5:**
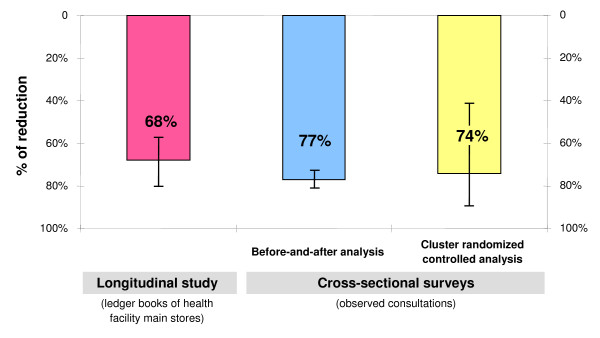
**Reduction (with 95%CI) of first-line anti-malarials consumption by all three assessments: **(1) longitudinal study based on ledger books of intervention health facility main stores, (2) before-and-after analysis of cross-sectional surveys of observed consultations and 3) cluster randomised controlled analysis of cross-sectional surveys of observed consultations.

The implementation of *m*RDTs for malaria in nine health facilities in Dar es Salaam in near-programme conditions led to a dramatic reduction in anti-malarials consumption. This was confirmed in all three methods of evaluation with two independent data sets: the longitudinal analysis of routine statistics (Post-over Pre-intervention ratio of 0.32 for ALu and 0.37 for injectable quinine), the before-and-after study based on pre- and post-intervention surveys (Risk ratio 0.23 for the first-line treatment and 0.35 for injectable quinine) and the cluster randomized analysis comparing matched intervention and control health facilities (Risk ratio 0.26 for the first line treatment and 0.43 for injectable quinine) (see Figure 5).

The high level of convergence of the results confirms the robustness to the findings. The two main reasons for this decrease were illuminated by the observations of patient-provider interactions in the repeated cross sectional surveys. Firstly, the higher accuracy of routine *m*RDTs compared to routine microscopy led to a dramatic reduction in the number of positive patients. Secondly, as health workers trusted *m*RDT results, the proportion of test-negative patients treated with anti-malarials dropped from 53% to 7%. The impact was maintained up to the end of the observation period (18 months) and even increased after the initial four months thanks to targeted programmatic actions in poor-performing HF. In the control HF there was a moderate decrease in ALu consumption (PP ratio 0.68), but a corresponding increase in quinine consumption and in the number of patients diagnosed with malaria.

The repeated cross sectional surveys showed that 1.49 *m*RDTs were needed to save one malaria treatment course. This was, however, at the cost of an additional 0.41 antibiotic treatment courses. If clinicians had been fully adherent to both patients selection for *m*RDT testing and treatment upon *m*RDT result, only 1.22 *m*RDTs would have been required. The post-intervention survey took place just after the rainy season, when 18% of patients complaining of fever were positive by *m*RDT. If the malaria prevalence had been 5% (the lowest monthly rate observed in Dar es Salaam), clinicians would have needed 1.05 *m*RDTs per anti-malarial treatment saved. From the longitudinal study, in which wastage of drugs between the main store and the dispensing window was important, it was found that 2.15 *m*RDTs were necessary to save one ALu blister and half a vial of quinine. These observations show clearly how circumstances shape the effectiveness of *m*RDT implementation.

An interesting observation was the "contamination" of the control HF with some of the key messages from the training activities. This was mainly due to health workers from intervention HF being transferred to control HF during the study period. The message that the incidence of malaria in Dar es Salaam was much lower than commonly thought was clearly passed on to control HF. This helped clinicians to withhold anti-malarials when the result of microscopy was negative and presumably also the microscopists to refrain from giving so many false positive results.

Routine statistics were useful in this case because of the reasonable quality of registers in Dar es Salaam. Besides giving a robust confirmation that clinicians' behaviour changes took place, this gave for the first time a unique measure of the amount of anti-malarial drug that could be saved following *m*RDT implementation: 12,727 ALu blisters and 6,061 quinine vials per month in nine HF, including the three district hospitals. The analysis of these routine statistics also identified another important source of drug wastage: the mishandling of drug stocks that were either lost, diverted or got expired between the main store and the patients. Initiatives aimed at reducing drug wastage should thus not only target clinicians' prescription behaviour but also drug management more generally.

There are currently nine studies that looked at the impact of *m*RDT on anti-malarial prescription or clinicians' adherence to *m*RDT result. One Kenyan study was inconclusive because adherence to test result was already very high prior to any intervention [19]; two studies from Tanzania and Burkina Faso showed no effect of mRDT at all [12,13]; three studies from Zanzibar [20], Uganda [21] and Tanzania mainland [22] showed a strong impact (RR 0.42 and 0.29 for anti-malarial prescription and RR 0.29 for over-prescription respectively). A Zambian survey, conducted one year after deployment of mRDT at national scale, showed intermediate results (RR 0.62 for negative patients treated with anti-malarials), but was underpowered because of an unexpected low number of patients tested for malaria [23]. A study in Ghana found a moderate impact in facilities without microscopy (RR 0.75 for anti-malarial prescription) and no impact at all in facilities using microscopy previously [14]. A recent study from Uganda showed a moderate impact (RR 0.62 for anti-malarial prescription) due to a significant number of patients still treated while negative [24]. All these studies are very heterogeneous in terms of setting, design and type of training and there are probably several reasons for failure or success. These studies were all different from the present one in several ways: they took place in rather controlled conditions (except the Zambian study), used consultation observations only, and were conducted shortly after the start of *m*RDT implementation (with or without a baseline survey). In Zambia, the very first experience of *m*RDT use in Africa outside South-Africa, the main problem was probably the assumption that clinicians would act upon *m*RDT result without problem [23]. By contrast, the successful Ugandan study put a strong emphasis on training and on giving straightforward messages [21]. The successful Zanzibar study used a weekly cross-over design where nurses received a financial incentive to participate in the study and adhere to specific instructions on mRDT [20]. The Tanzanian mainland study was also successful although no direct incentive was given, but a study staff member was physically on-site during the entire study period and facilities were visited frequently by the supervisory staff [22]. The reasons given by the authors of the Tanzanian study showing no impact was the insufficient training on *m*RDT given to clinicians [12]. Another possible explanation for the low impact of *m*RDT implementation found in this study, as well as in the studies of Burkina Faso [13] and Ghana [14] might be related to the study methodology: they all used the patient as allocation/randomization unit and not the health facility. However, introducing *m*RDT requires an in-depth change of the provider's diagnosis and treatment concepts. This can only be achieved with a supporting environment, which constantly and consistently re-enforces the new approach. Such a change cannot be expected when the same clinician is asked to apply two different strategies to the patients s/he sees during the same day. The only way to significantly improve the outcome of RDT implementation is when all professionals working in one health facility consistently implement one strategy.

Based on the observations made during the study and on the feed-back given by clinicians, it is postulated that the major determinants for the positive results of the present programme were the following: (1) the study was presented to clinicians as the pilot phase of a planned national deployment of *m*RDT; (2) one of the main investigator of the study was the City Medical Officer of Health, hence health workers did not consider this study as an isolated research project but rather as the new guidelines for malaria management in the city; (3) the training and the evidence it provided was appreciated by the target audience of health care professionals. For them, the evidence on the low malaria prevalence in Dar es Salaam, on the bad quality of routine microscopy and on the excellent performance of *m*RDT (from meta-analyses and data acquired locally) was new and very relevant. They also had the possibility to link this knowledge to their own reality with cases studies. The health care workers also appreciated the possibility to discuss the mutual mistrust between laboratory staff and clinicians with regard to malaria testing, and that they were presented a tool (the *m*RDTs) to overcome this issue. Clear take-home messages were also given, avoiding ambiguous messages [25]. It is likely that the present experience would be reproducible in the context of a well-planned programmatic deployment of *m*RDT in the public health system. Indeed this programme included only a short training (one-day), limited supervision (once quarterly) and absence of financial incentives. With regard to the applicability of these results in other settings, we carried out a similar study in a rural area of Tanzania (Kilombero/Ulanga Districts), although without a control group [26]. The endemicity there was higher than in Dar es Salaam. The impact on the overall consumption of anti-malarials was, therefore, less (a two-fold decrease) due to the much higher proportion of fever cases associated with parasitaemia (39% instead of 8% in Dar es Salaam). Interestingly, the adherence of clinicians to the recommendation of not treating negative patients was even higher in that remote setting (only 1% of negative patients received an anti-malarial drug), similarly to what was observed recently with community health workers using *m*RDT in Zambia [27].

Besides saving anti-malarial treatments, one important aspect of RDT implementation is the selection of patients for malaria testing. Patients presenting with a wide range of medical condition were deliberately included to explore clinicians' behavior towards malaria test requests. During the training, the necessity of testing only patients complaining of fever was emphasized. Unfortunately no impact was obtained on this outcome. During the feed-back meeting organized after the end of the study, clinicians stated that the pressure of patients for testing (much more than treating) was high, in particular when coming for a check-up. Moreover, in contrast to treatment, there is no risk for the patient to be tested.

The large-scale deployment of mRDT is of course going beyond behaviour change alone. There are major health system challenges, starting with the need to considerably strengthen the capacity to order/purchase and deliver goods (*m*RDT and drugs) in areas that have always had poor coverage of health interventions [28]. The respective role of mRDT and/or microscopy needs to be determined in different settings and different clinical situations. Also, an effective quality assurance system to support *m*RDT introduction needs to be established. Some major improvements have been made to develop a monitoring process to evaluate the performance of different brands and batches of *m*RDT in recent years [29]. Considerable efforts need still to be invested to ensure appropriate use of *m*RDT in the field, with a focus on the integrated management of childhood illness in the community and the management of non-malaria fevers. An increased use of broad-spectrum antibiotics in most of *m*RDT negative cases has indeed been shown in the present study (in the cross-sectional surveys but not in the longitudinal study probably because the effect of RDT diluted itself in the overall very large consumption of antibiotics by HFs for all types of patients) as well as in recent studies in Tanzania and Zanzibar [20,30]. A significant increase in the indiscriminate use of antibiotics in sub-Saharan Africa is likely to add to the global problem of antibiotic resistance and should be prevented by all means [31].

### Limitations of the study

The main limitation of the cluster randomized control study is the low number of clusters (three). When the study was designed, the priority was deliberately put on the before-and-after component. Indeed, the high heterogeneity between HF makes them quite difficult to be compared, even after matching on several criteria. Contamination between intervention and control HF was also a risk that in fact occurred during this study. Also knowing the potential benefit of RDT, to convince more health facilities to be in the control arm would have been difficult. Knowing that the malaria transmission would not have time to change drastically in a 2 year period, the before-and-after analysis was thus considered to be potentially more robust than the cluster randomized control analysis.

Another limitation comes from the longitudinal study in which the impact of *m*RDT on anti-malarials consumption on the long term might have been underestimated. Indeed the ALu decrease was less pronounced in the first 4 months post *m*RDT initiation than in the following months. This was related to problems at the start of the implementation - which could be solved. Also the total number of attendances slightly increased over time, while the data for tests and drugs were not corrected by the total number of patients. The pre-intervention period was very short for ALu (3 months), which reduces the strength of the assessment. However, the impact measured on the consumption of quinine vials, which was based on a longer pre-intervention period (15 months), was quite similar to that of ALu.

## Conclusions

When deployed appropriately (official support of the new tool by senior health authorities, high quality training, regular routine supervision and monitoring after implementation), *m*RDTs lead to a considerable saving of oral and injectable anti-malarial drugs at all levels of the health system, including in hospitals. *m*RDT also prevent patients from misdiagnosis and adverse events of unnecessary anti-malarial treatments. The potential of anti-malarial saving through *m*RDT use could be maximized if other causes of drug wastage were tackled as well, i.e. drug procurement mechanisms based on the number of confirmed malaria patients, rigorous and dynamic management of stocks, similar diagnostic and treatment strategies in the private sector including pharmacies and drug shops [32]. The downside of *m*RDT implementation is the shift from anti-malarial wastage to antibiotic wastage due to insufficient knowledge and training on other causes of fever. Deployment of *m*RDT should therefore move hand in hand with strategies aimed at reducing irrational use of antibiotics at outpatient level, for example through updated IMCI decision charts promoted by innovative approaches for teaching and communication.

## Conflict of interests

All authors have completed the Unified Competing Interest form at http://www.icmje.org/coi_disclosure.pdf (available on request from the corresponding author) and declare (1) No support received from any company for the submitted work; (2) No financial relationships with commercial entities that might have an interest in the submitted work; (3) No spouses, partners, or children with relationships with commercial entities that might have an interest in the submitted work; and (4) No non-financial interests that may be relevant to the submitted work.

## Authors' contributions

VDA, BG and CL designed the study. VDA and JKM led the project in the field. NS helped with the training and supervision of health workers. DM acted as facilitator to conduct the study in the field. VDA analysed the data and wrote the manuscript. BG, CL and JKM contributed to the manuscript. All authors commented on the paper and agreed on the content.
